# Verapamil extends lifespan in *Caenorhabditis elegans* by inhibiting calcineurin activity and promoting autophagy

**DOI:** 10.18632/aging.102951

**Published:** 2020-03-24

**Authors:** Wenwen Liu, Huiling Lin, Zhifan Mao, Lanxin Zhang, Keting Bao, Bei Jiang, Conglong Xia, Wenjun Li, Zelan Hu, Jian Li

**Affiliations:** 1 State Key Laboratory of Bioreactor Engineering, Shanghai Key Laboratory of New Drug Design, East China University of Science and Technology, Shanghai, China; 2 Institute of Materia Medica, Dali University, Dali, China; 3 College of Pharmacy and Chemistry, Dali University, Dali, China; 4 National Institute of Biological Sciences, Beijing, China

**Keywords:** verapamil, *Caenorhabditis elegans*, anti-aging, cell senescence, autophagy

## Abstract

Previous evidence has revealed that increase in intracellular levels of calcium promotes cellular senescence. However, whether calcium channel blockers (CCBs) can slow aging and extend lifespan is still unknown. In this study, we showed that verapamil, an L-type calcium channel blocker, extended the *Caenorhabditis elegans* (*C. elegans*) lifespan and delayed senescence in human lung fibroblasts. Verapamil treatment also improved healthspan in *C. elegans* as reflected by several age-related physiological parameters, including locomotion, thrashing, age-associated vulval integrity, and osmotic stress resistance. We also found that verapamil acted on the α1 subunit of an L-type calcium channel in *C. elegans*. Moreover, verapamil extended worm lifespan by inhibiting calcineurin activity. Furthermore, verapamil significantly promoted autophagy as reflected by the expression levels of LGG-1/LC3 and the mRNA levels of autophagy-related genes. In addition, verapamil could not further induce autophagy when *tax-6*, calcineurin gene, was knocked down, indicating that verapamil-induced lifespan extension is mediated via promoting autophagy processes downstream of calcineurin. In summary, our study provided mechanistic insights into the anti-aging effect of verapamil in *C. elegans*.

## INTRODUCTION

Aging essentially involves physiological decline that leads to impaired function and promotes mortality [1]. It is the major cause of several human diseases, such as neurodegenerative and cardiovascular disorders and type 2 diabetes [2, 3]. Although aging is inevitable, the pace of aging can be modulated and regulated to a certain extent. In recent decades, several studies have revealed the hallmarks of aging (e.g., loss of proteostasis, mitochondrial dysfunction, and telomere attrition) and the associated signaling pathways (e.g., insulin/IGF-1, mTOR, AMPK, and germline signaling pathways) [4–10]. Based on these findings, novel anti-aging agents have been developed to target multiple signaling pathways by either activating or inhibiting certain intermediate proteins [11]. These candidate agents include senolytic drugs (dasatinib and quercetin), mTOR inhibitors (rapamycin), AMPK activators (metformin), sirtuin activators (resveratrol), and so on [12–15]. Some of these drugs have shown promising potential in promoting longevity and have entered the clinical trial stage [16].

With an aim to discover candidate anti-aging compounds, we have screened 1,386 FDA-approved drugs using *C. elegans* as an animal model for the evaluation of lifespan extension. We obtained several hit compounds that exhibited significant effects on lifespan extension. Here, verapamil (C_27_H_38_N_2_O_4_·HCl) was selected as the candidate anti-aging compound (Figure 2A). Verapamil, a first-generation CCB, has been widely employed for the treatment of hypertension. Recent findings also showed that verapamil treatment is associated with reduced fasting glucose levels in the serum of adult diabetes patients and along with reduced incidence of diabetes mellitus [17, 18]. In mouse models of diabetes, verapamil administration enhanced the endogenous levels of insulin, ameliorated glucose homeostasis, and reduced β-cell apoptosis [19]. In addition, verapamil is also involved in the regulation of cardiac gene transcription and chromatin modification via a novel calcineurin-nuclear factor Y signaling pathway [20].

**Figure 1:**
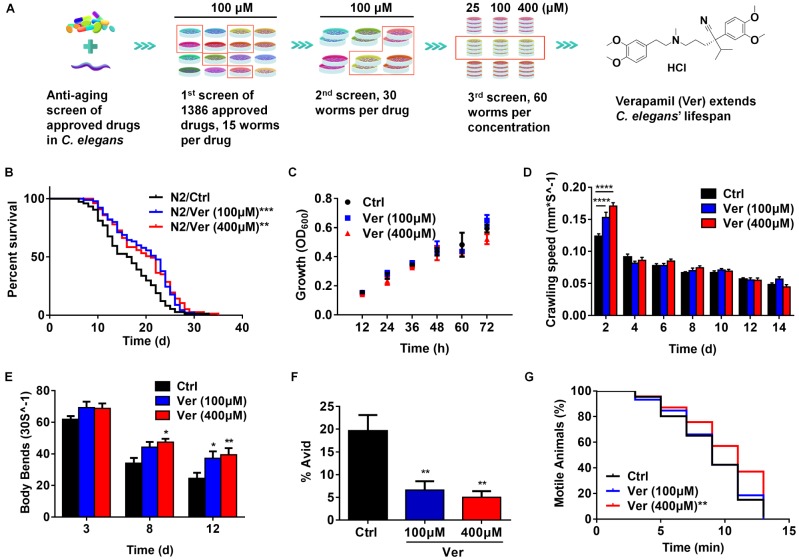
**Verapamil extends lifespan and improves healthspan in *C. elegans*.** (**A**) Around 1,386 FDA-approved drugs were screened, and *C. elegans* were used as the model for lifespan evaluation. Finally, verapamil was selected as a hit anti-aging compound. (**B**) Verapamil extended the lifespan of wildtype *C. elegans* (N2) at 100 μM (^***^P < 0.001) and 400 μM (^**^P < 0.01). (**C**) Verapamil (100 μM and 400 μM) did not reduce bacterial growth. Multiple t-tests were used to calculate the *P*-values and error bars represent SEM. (**D**) Verapamil increased the crawling speed of worms on day 2 (100 μM, ^****^P < 0.0001; 400 μM, ^****^P < 0.0001), but had no influence in late life. (**E**) Verapamil significantly increased the number of body bends on day 8 (400 μM, ^*^P < 0.05) and day 12 (100 μM, ^*^P < 0.05; 400 μM, ^**^P < 0.01). A two-way ANOVA along with Sidak multiple comparisons test was used to calculate *P*-values, and error bars represent SEM in (**D**) and (**E**). (**F**) Total Avid was significantly decreased by verapamil (100 μM, ^**^P < 0.01; 400 μM, ^**^P < 0.01). An unpaired t-test was used to calculate the *P*-values and error bars represent SEM. (**G**) Verapamil specifically improved the resistant to osmotic stress (400 μM, ^**^P < 0.01), but had no effect at 100 μM. The log-rank (Mantel-Cox) test was used to assess the *P*-values in (**B**) and (**G**).

**Figure 2:**
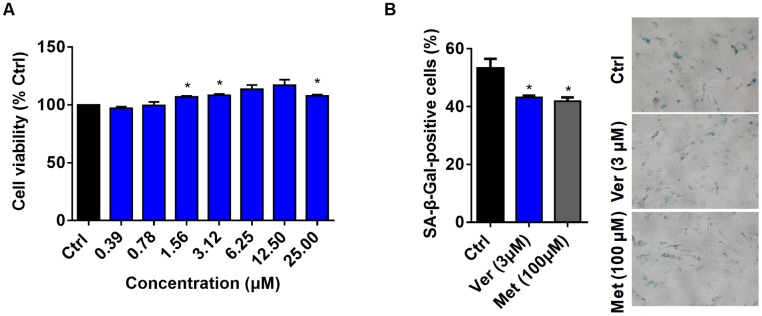
**Verapamil enhances cell viability and delays cellular senescence.** (**A**) Viability of MRC-5 cells in the absence (Ctrl) or presence of verapamil (Ver) at different concentrations. (**B**) SA-β-Gal staining of MRC-5 cells and quantification of SA-β-Gal-positive cells at a late passage (P31). Verapamil (3 μM) delayed the senescence of MRC-5 cells (^*^P < 0.05). Metformin (100 μM) was used as positive control (^*^P < 0.05). An unpaired t-test was used to calculate the *P*-values and error bars represent SEM.

To date, verapamil has not been reported to exhibit anti-aging effects. Since verapamil acts as a CCB, we first focused on the calcium signaling involved in cellular senescence. Accumulating evidence has revealed that upregulation of intracellular levels of calcium promotes cellular senescence [21]. The cells can be rescued from senescence via knockdown of IPR3 channel, which is involved in calcium release, and chelation of calcium with BAPTA [22, 23]. However, the calcium channels that are involved in the modulation of cellular senescence remain largely unknown [24]. Increase in the cytosolic levels of calcium promotes the binding of calcium to a calcium sensory protein, calmodulin, which triggers calcineurin activation that acts as a critical connecting link between calcium signaling and longevity [25]. Previous studies have also revealed *in vitro* anti-senescence effects of some CCBs. Isradipine can attenuate rotenone-induced senescence in human neuroblastoma SH-SY5Y cells [26]. Nifedipine can prevent high glucose-induced senescence in human umbilical vein endothelial cells [27]. However, whether CCBs can slow aging and extend lifespan is still unknown. Here, we investigated the biological effects of verapamil in *C. elegans* and human lung fibroblasts and studied the lifespan-extending mechanism of verapamil.

## RESULTS

### Verapamil extends lifespan and improves healthspan in *C. elegans*

After three rounds of screening approved drugs, we obtained hit compounds that extended the lifespan of *C. elegans* (Figure 1A). Treatment with 100 μM and 400 μM verapamil led to an average lifespan extension of 20.59% and 19.45%, respectively, compared to the control group (Figure 1B, Table 1). To examine if verapamil treatment extended *C. elegans* lifespan via bacterial growth inhibition, we assessed bacterial growth. However, bacterial growth was not inhibited by verapamil treatment (Figure 1C).

**Table 1:** Lifespan data.

**Strain**	**Drug treatment**	**RNAi**	**Mean lifespan (days)**	**Maximum lifespan (days)**	**Number of worms**	***P*-Values**
N2	—	—	16.52	33	74	—
N2	Ver (100μM)	—	19.92	31	90	< 0.001
N2	Ver (400μM)	—	19.71	33	77	< 0.01
N2	—	—	15.37	22	197	—
N2	Ver (100μM)	—	17.94	26	196	< 0.0001
N2	Ver (400μM)	—	17.15	24	193	< 0.0001
*bec-1(ok700)*	—	—	13.70	21	92	—
*bec-1(ok700)*	Ver(100μM)	—	13.37	21	176	0.0546
N2	—	—	13.24	24	171	—
*egl-19(n582)*	—	—	9.16	22	188	—
*egl-19(n582)*	Ver (100μM)	—	9.18	20	133	0.6855
*egl-19(n582)*	Ver (400μM)	—	9.45	22	153	0.2613
N2 AL	—	—	14.86	23	52	—
N2 DR	—	—	19.11	28	101	—
N2 DR	Ver (400μM)	—	20.09	31	53	< 0.05
N2	—	L4440	12.54	17	166	—
N2	Ver (100μM)	L4440	14.24	22	161	< 0.0001
N2	—	*tax-6*	15.44	25	159	—
N2	Ver (100μM)	*tax-6*	15.68	25	152	0.0713

Since frailty is the most problematic expression of population aging, it is important to test whether anti-aging compounds reduce severity of frailty and improve healthspan [28–30]. Therefore, we assessed the effects of verapamil on several age-related physiological parameters. First, we evaluated the crawling speed and found that verapamil-treated groups exhibited higher locomotive activity at day 2 (Figure 1D). Next, we assessed the effects of verapamil on the motility of *C. elegans* by analyzing the body bend rate. The numbers of body bends every 30 seconds were counted on day 3, day 8, and day 12 of adulthood. The verapamil-treated groups exhibited a more intense swinging motion compared to the untreated group (Figure 1E). Furthermore, we evaluated the age-associated vulval integrity (Avid), which is a useful measurement of healthspan in worm populations [31]. Our results showed that verapamil-treated worms exhibited a significant decrease in Avid (Figure 1F). Furthermore, osmotic stress response was evaluated to assess the survival extension ability under hyperosmotic environments. We found that verapamil (400 μM)-treated group exhibited significant improvement in worms’ survival; however, treatment with 100 μM verapamil did not lead to any positive effect (Figure 1G). In addition, verapamil (100 μM and 400 μM) treatment had no significant effect on heat stress tolerance (Supplementary Figure 1). Therefore, these results demonstrated that verapamil prolongs the lifespan and improves age-related physiological parameters**.**

### Verapamil enhances cell viability and delays cellular senescence

Cellular senescence is closely related to age-related diseases [32]. The senescent cells secrete several proinflammatory proteases, chemokines, cytokines, etc. This phenomenon is known as senescence-associated secretory phenotype (SASP) [33]. In 1995, Dimri et al. demonstrated that senescence-associated β-galactosidase (SA-β-Gal) is a good biomarker of cellular senescence; its activity is detected by 5-bromo-4-chloro-3-indolyl β-D-galactosidase (X-gal), which forms a blue precipitate upon cleavage [34]. It has also been previously shown that the senescence of human dermal fibroblasts could be delayed by treatment with the anti-aging drug candidate, metformin [35].

Therefore, we investigated the anti-aging effects of verapamil on mammalian cells. First, to select the optimal concentration of verapamil for human lung fibroblast MRC-5 cells, we treated these cells with varying concentrations of verapamil for 3 days and assessed the viability of these cells using a CCK8 kit. The results showed that verapamil had a tendency to promote cell viability at concentrations of up to 25 μM (Figure 2A). Then, we examined whether 3 μM verapamil could delay cellular senescence. Cells were treated for 3 days with verapamil and stained them with X-gal. Metformin-treated cells were used as the positive control. The proportion of cells positive for SA-β-Gal was significantly reduced in the groups treated with verapamil or metformin compared to the control group (Figure 2B), which indicated that verapamil treatment can adversely affects the senescence of MRC-5 cells. Taken together, these results indicated that low-dose verapamil treatment increased human cell viability and delayed senescence.

### Verapamil acts on calcium channels in *C. elegans*

**Figure 3:**
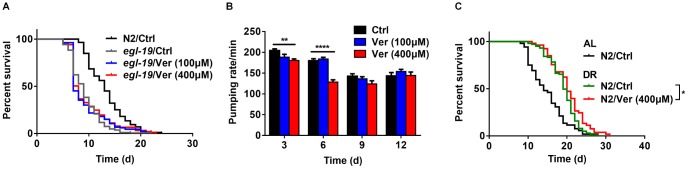
**Verapamil acts on the α1 subunit of an L-type calcium channel in *C. elegans*.** (**A**) Verapamil (100 μM and 400 μM) did not extend the lifespan of *egl-19* mutant worms expressing a defective L-type Ca^2+^ channel α1 subunit. (**B**) Verapamil (400 μM) decreased the pharyngeal pumping rate, especially at day 3 and 6; however, 100 μM verapamil had no effect on the pumping rate. A two-way ANOVA along with Sidak multiple comparisons test was used to calculate the *P*-values and error bars represent SEM. (**C**) Verapamil (400 μM) extended the lifespan even under bacterial dilution conditions (^*^P < 0.05). The log-rank (Mantel-Cox) test was used to assess the *P*-value in (**A**) and (**C**).

Voltage-activated calcium channels contain a pore-forming α1 subunit, which is associated with up to three auxiliary subunits (*α*2/*δ*, *β*, and *γ*). These channels are classified into L-, N-, P-, Q-, R-, and T-types based on the Ca^2+^ current conductance [36]. Verapamil blocks L-type calcium channels by binding with their α1 subunit and mediates its function via the calmodulin and CaM kinase pathway in mammalian cells [37–39]. In *C. elegans*, the α1 subunit of voltage-activated L-type calcium channel is encoded by the *egl-19* gene [40]. Based on the above studies, we wanted to determine whether verapamil acts on calcium channels of *C. elegans*. Since loss-of-function mutations in *egl-19* completely abolish muscle contraction, which leads to death, a mutant with downregulated *egl-19* expression was selected for lifespan assay [40]. We found that verapamil could not extend this mutant’s lifespan (Figure 3A and Table 1). This result indicated that the effect of verapamil on the worm’s lifespan could mediate through the α1 subunit of its L-type calcium channel.

In *C. elegans*, the action potential of the pharyngeal muscle partly depends on its L-type calcium channel. Increase in the extracellular levels of Ca^2+^ leads to a higher action potential, whereas this action potential decreases on treatment with the L-type calcium channel blockers, like verapamil [41]. We measured the pharyngeal pumping rate (Figure 3B) and found that treatment with 400 μM verapamil reduced the pharyngeal pumping rate significantly on days 3 and 6, while treatment with 100 μM verapamil did not. Since *C. elegans* feeds through its pharynx, decreasing pharyngeal pumping rate might result in dietary restriction (DR). During treatment with 400 μM of verapamil, a DR mechanism might be involved in the extension of *C. elegans* lifespan. We, therefore, investigated the effects of verapamil on worm lifespan under DR conditions. Treatment with 400 μM verapamil extended the lifespan even under DR conditions (Figure 3C, Table 1), suggesting that verapamil, at such high dose, might extend the lifespan not only via a DR mechanism but also via other mechanisms. Since treatment with 100 μM verapamil had no effect on the pharyngeal pumping rate, we studied the mechanism of 100 μM verapamil's action in further sections.

### Verapamil extends *C. elegans* lifespan by inhibiting calcineurin activity

Intracellular calcium usually exerts its biological effects via calmodulin-calcineurin pathway [37]. The main mechanisms by which Ca^2+^ acts is by binding to and activating calmodulin, Ca^2+^-binding proteins, and other major intracellular receptors [42]. Calcineurin is a Ca^2+^-calmodulin-dependent serine/threonine protein phosphatase that is a crucial component of several signaling pathways [43].

**Figure 4:**
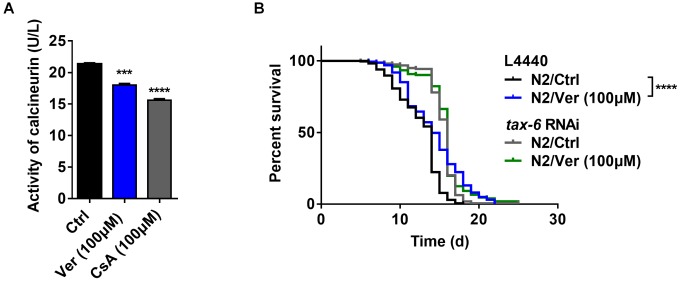
**Verapamil extends lifespan by inhibiting the activity of calcineurin.** (**A**) Verapamil (100 μM) reduced the activity of calcineurin in *C. elegans* (^***^P < 0.001). Cyclosporin A (CsA) was used as positive control (^***^P < 0.001). An unpaired t-test was used to calculate the *P*-values and error bars represent SEM. (**B**) Verapamil (100 μM) did not extend the lifespan of worms treated with *tax-6* RNAi. The log-rank (Mantel-Cox) test was used to assess the *P*-values.

In *C. elegans*, calcineurin is composed of a regulatory subunit (calcineurin B, encoded by *cnb-1*) and a catalytic subunit (calcineurin A, encoded by *cna-1/tax-6*) [44, 45]. First, we identified, via ELISA, whether blocking calcium channels with verapamil inhibited calcineurin activity. We found that verapamil significantly inhibited calcineurin activity in *C. elegans* (Figure 4A). Then, we investigated the effects of verapamil over worm lifespan using worms (N2) fed with bacteria engineered to produce *tax-6* RNAi effects. The efficacy of RNAi in decreasing *tax-6* mRNA levels was assessed using qPCR (Supplementary Figure 2). Compared to worms fed with bacteria expressing the control vector (L4440), the worms with *tax-6* RNAi significantly extended the lifespan of worms (Figure 4B, Table 1), which was consistent with the findings of a previous study [46]. When *tax-6* expression was knocked down, verapamil no longer extended the lifespan of worms (Figure 4B, Table 1). In summary, verapamil inhibits the activity of calcineurin and extends the lifespan via a calcineurin-dependent mechanism.

### Verapamil extends *C. elegans* lifespan by activating autophagy

**Figure 5:**
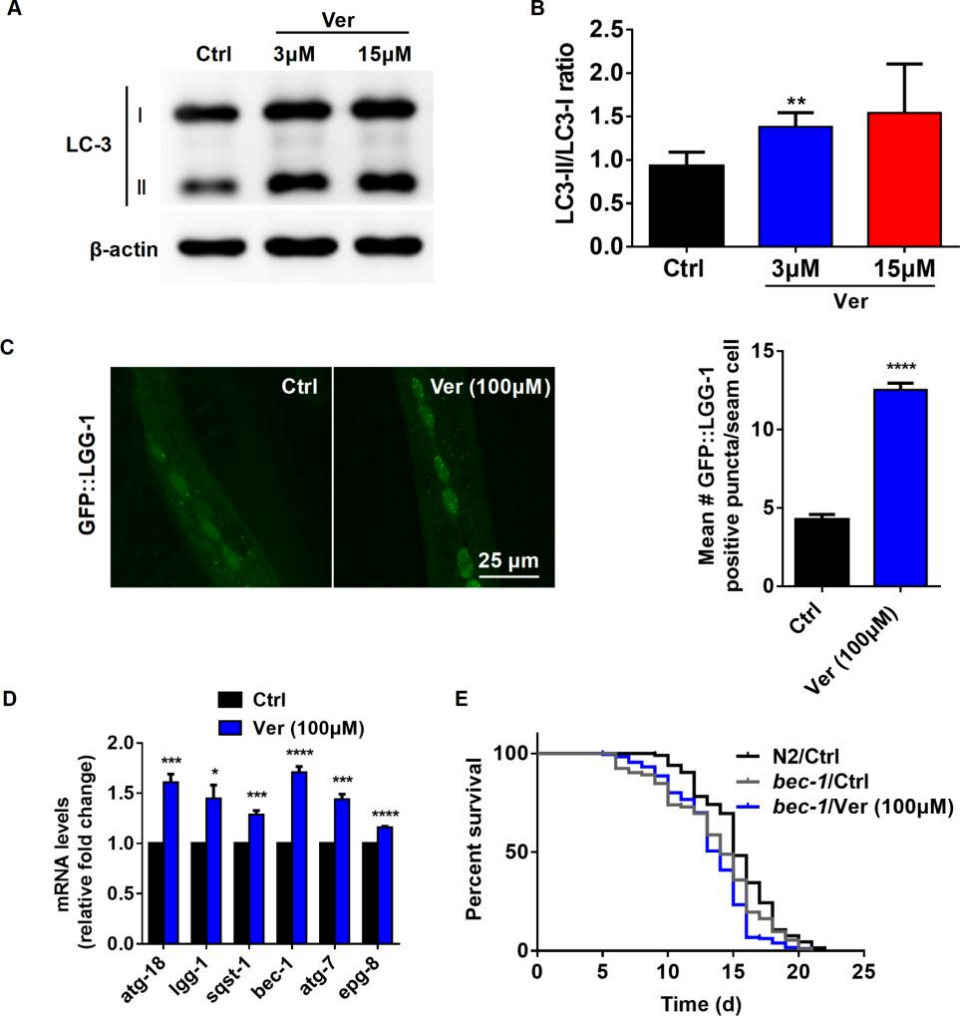
**Verapamil extends lifespan through activating autophagy in *C. elegans*.** (**A**, **B**) The degree of autophagy induced by verapamil (3 μM and 15 μM) was evaluated by assessing the LC3-II/LC3-I ratio in MRC-5 cells. (**C**) GFP::LGG-1 levels were evaluated in the seam cells during the L3 stage (^****^P < 0.0001) to assess the degree of verapamil (100 μM)-induced autophagy. (**D**) Verapamil (100 μM) significantly activated the expression of autophagy-related genes. Multiple t-tests were used to evaluate the *P*-values and error bars represent SEM. (**E**) Verapamil (100 μM) did not extend the lifespan of worms in which *bec-1* expression was downregulated. The log-rank (Mantel-Cox) test was used to calculate the *P*-value.

Although Ca^2+^ signaling has been shown to be involved in several fundamental cellular processes, its role in autophagy is still somewhat ill-defined [47]. It has previously been reported that cytosolic Ca^2+^ signals can exhibit both pro- and anti-autophagic effects [48]. Therefore, we investigated whether verapamil facilitates autophagy in human cells and *C. elegans*. First, autophagy induced by verapamil was assessed by measuring the LC3-II/I ratio in MRC-5 cells (Figure 5A, 5B) and by quantifying the autophagic vesicles in hypodermal seam cells of GFP::LGG-1 L3 larvae (Figure 5C). Moreover, we found that verapamil treatment increased the expression of several autophagy-related genes, including *atg18*, *lgg-1*, *sqst-1*, *bec-1*, *atg-7*, and *epg-8*, leading to enhanced autophagy in *C. elegans* (Figure 5D).

Then, to confirm whether autophagy induction is relevant to verapamil-mediated extension of *C. elegans* lifespan, we assessed the lifespan of *bec-1(ok700)* mutant, in which autophagy mediator BECLIN was downregulated. We found that verapamil no longer extended *bec-1(ok700)* mutant lifespan (Figure 5E, Table 1). Altogether, these results suggested that verapamil extended worm lifespan by activating autophagy.

### Verapamil facilitates autophagy downstream of calcineurin in *C. elegans*

**Figure 6:**
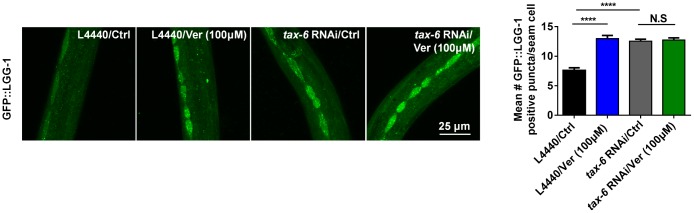
**Verapamil facilitates autophagy downstream of calcineurin in C. elegans.** *tax-6* RNAi induced autophagy in worms expressing GFP::LGG-1 (^****^P < 0.0001); however, verapamil (100 μM) did not facilitate autophagy under *tax-6* RNAi treatment. An unpaired t-test was used to calculate the *P*-values and error bars represent SEM.

As reported previously, calcineurin loss-of-function/null mutants, which are long-lived, exhibited enhanced autophagy than gain-of-function mutants and wild-type worms. However, when treated with RNAi against pro-autophagic gene, the lifespan of the calcineurin mutant lost its extended lifespan phenotype and enhanced autophagy. These results suggested that autophagy-related genes are required for lifespan extension in calcineurin-defective worms [25]. Since verapamil inhibits the activity of calcineurin and facilitates autophagy, we evaluated whether autophagy is linked with calcineurin activity in verapamil-mediated lifespan extension. We fed GFP::LGG-1 worms with bacteria engineered to produce *tax-6* RNAi effects. We found that, compared with control vector (L4440) treatment, treatment of worms with either *tax-6* RNAi or verapamil significantly facilitated autophagy (Figure 6). In addition, verapamil treatment did not further upregulate GFP::LGG-1 expression in worms treated with *tax-6* RNAi (Figure 6). In summary, verapamil inhibited the activity of calcineurin and did not promote autophagy in worms treated with *tax-6* RNAi, indicating that verapamil triggers autophagy by inhibiting the activity of calcineurin.

## DISCUSSION

In recent decades, an array of strategies has appeared and has been experimented upon under the ‘anti-aging’ umbrella. Several signaling pathways related to either aging or longevity have been reported and provided guidance to research studies [11]. The development of new anti-aging drugs seems to be an immense opportunity for the pharmaceutical and healthcare industries [49]. We devoted attention to repurposing approved drug and obtained some hit compounds. In this study, we selected verapamil as a candidate anti-aging agent among 1,386 FDA-approved drugs. We showed that verapamil induces lifespan extension in *C. elegans* and promotes physiological parameters including locomotion, thrashing, age-associated vulval integrity, and osmotic stress. In addition, verapamil treatment extended the lifespan of *Drosophila melanogaster (D. melanogaster)* (Supplementary Figure 3, Supplementary Table 1) and delayed senescence in MRC-5 cells, suggesting that it could exhibit potential anti-aging effects in different organisms.

Next, we found that verapamil failed to extend the lifespan of worms with α1 subunit (*egl-19*) mutation, suggesting that verapamil acts on the α1 subunit of the L-type calcium channel in *C. elegans*, which was consistent with the results in mammals. We speculated that blocking this membrane calcium channel by verapamil decreased the intracellular Ca^2+^ concentration, which led to a reduction in calmodulin activity. Then, we found that verapamil decreased the activity of calcineurin in *C. elegans* and no longer extended worm lifespan after *tax-6* RNAi treatment. These results indirectly indicated that verapamil inhibits calcineurin activity by modulating the calcium concentration. Given the important role of Ca^2+^ signals in autophagy, the expression of LC3/LGG-1, and the mRNA levels of autophagy-related genes were tested. These results showed that verapamil promotes autophagy. In addition, verapamil no longer extended the lifespan of *bec-1(ok700)* mutant, which means that activation of autophagy is relevant to verapamil*-* mediated lifespan extension. Since the calcineurin- related lifespan extension is closely related to autophagy [25], we investigated the relationship between calcineurin activity and autophagy under verapamil treatment. We found that verapamil failed to promote autophagy in the *tax-6* RNAi background. Overall, the results indicated that verapamil extends *C. elegans* lifespan via inhibition of calcineurin and autophagy induction.

We obtained the above results at a verapamil concentration of 100 μM. At 400 μM, verapamil significantly decreased the pharyngeal pumping rate, which led to some extent of dietary restriction in *C. elegans*. Therefore, at high concentrations, verapamil may extend the lifespan via two or more mechanisms, which need further in-depth study. In addition, calcium signaling has been reported to regulate several biological functions in addition to cellular senescence. Here, we discussed the probable cause-and-effect relationship between autophagy induction and calcineurin activity in *C. elegans*; however, possible molecular mechanisms linking the autophagic pathway and calcineurin activity remain to be investigated. To further explore how verapamil regulates autophagy through the calcineurin pathway, we analyzed DAF-16/FOXO, a transcription factor related to regulation of aging [50]. We found that verapamil increased the lifespan of *daf-16* loss-of-function mutant, which indicated that verapamil’s mechanism of action is independent of DAF-16. Besides, verapamil did not upregulate the mRNA levels of DAF-16-specific targets, such as *sod-1*, *sod-2*, *sod-3*, *sod-4*, and *sod-5*, in wild-type worms (Supplementary Figure 4A, 4B, Supplementary Table 2). In addition, we did not observe any DAF-16::GFP nuclear translocation in cells treated with verapamil (Supplementary Figure 4C). Previously, the mammalian transcription factor EB (TFEB) orthologue, HLH-30, has been shown to modulate autophagy and longevity in *C. elegans* [51]. We found that verapamil treatment still increased the lifespan of the *hlh-30* knockout mutant and it didn’t produce any detectable HLH-30::GFP nuclear translocation (Supplementary Figure 5, Supplementary Table 2). Taken together, these results indicated that the action of verapamil on worm longevity is independent of DAF-16 and HLH-30. The pathway that links calcineurin inhibition to autophagy induction is still unknown.

Overall, our study was the first to show that the FDA-approved drug, verapamil, exhibits anti-aging effects in *C. elegans* and mammalian cells, and we studied its lifespan-extending mechanism in *C. elegans*. Our data suggested that verapamil extends *C. elegans* lifespan by inhibiting calcineurin activity and activating autophagy. In the context of global population aging, our work provides a new strategy for the discovery of anti-aging drugs, and broadens the application prospects of CCBs in the anti-aging field.

## MATERIALS AND METHODS

### *C. elegans* maintenance and strains

Strains were cultured on nematode growth medium (NGM) agar plates at 20 °C. We used wild-type N2 strain as reference. *C. elegans* strains used in this study included (name, genotype and origin): MT1212, *egl-19(n582) IV*, CGC; VC424, *bec-1(ok700) IV/nT1 [qIs51] (IV;V),* CGC; DA2123, *adIs2122 [lgg-1p::GFP::lgg-1 + rol-6(su1006)]*, CGC; CF1038, *daf-16(mu86) I*, CGC; TJ356, *zIs356 [daf-16p::daf-16a/b::GFP + rol-6(su1006)]*, CGC; MAH235, *sqIs19 [hlh-30p::hlh-30::GFP+ rol-6 (su1006)]*, CGC; *hlh-30 (hq293)* that was generated using CRISPR/Cas9 technology at Mengqiu Dong’s lab.

### Lifespan analysis

Lifespan analyses were conducted using the live *Escherichia coli* strain OP50 as food source. Worms were synchronized with bleaching buffer and maintained on NGM. Verapamil (Adamas, CAS: 152-11-4, 100 μM or 400 μM) was added to NGM. Plates were seeded with OP50. Worms were transferred at L4 stage to either control or verapamil (100 μM and 400 μM) treatment groups, with approximately 15-20 worms per 35-mm plate on day 0. In addition, 50 μg/mL of 5-Fluorodeoxyuridine (FudR) was added to the agar plates from day 0 to day 10 to avoid progeny hatching. Worms were counted every day and transferred to fresh plates every 3 days until all worms were dead. Worms that had either crawled off the plate or exhibited exploded vulva phenotype were censored. On day 10, all groups were transferred to control plates. Worms were treated with verapamil only for 10 days. Three replicate experiments were conducted. The survival curves were generated using GraphPad Prism 6. The log-rank (Mantel-Cox) test was used to assess curve significance.

### Bacterial growth assay

Bacterial growth assay was conducted as described previously [52]. A single colony of bacteria was inoculated in LB media and cultured at 37 °C. For plate assay, 30 μL of bacterial culture (OD_600_ = 0.4) was transferred to an NGM plate either or not containing verapamil (100 μM and 400 μM), and cultured at 20 °C. The bacteria were washed off using 1 mL M9 buffer and OD_600_ was measured every 12 h, with M9 buffer as the blank control. OD was assessed using a Hitachi U-2910 spectrometer with a 10-mm quartz cuvette. Three replicate experiments were conducted, and results were generated using GraphPad Prism 6. Unpaired t-test was used to assess the significance.

### *C. elegans* locomotion assay

Worms were grown after a lifespan assay, and a locomotion assay was conducted as described previously [53]. Locomotion was assessed every two days until day 14. A total of 20-30 worms per group were prepared for the control and verapamil (100 μM and 400 μM) treatment groups. Videos were captured, and locomotion was assessed using 30 s video of the worms’ movements by wrMTrck (ImageJ). The assay was repeated at least three times. Two-way ANOVA along with Sidak multiple comparisons test was used to evaluate the *P*-values.

### *C. elegans* thrashing assay

Worms were grown after a lifespan assay, and a thrashing assay was conducted as described previously [54]. For the control and verapamil (100 μM and 400 μM) treatment groups, thrashes were counted on days 3, 8, and 12. Any change in the midbody bending direction was referred to as a thrash [55]. First, one worm was removed and placed in an M9 buffer drop on an NGM plate without OP50 bacteria and allowed to adapt for 30 s. Then, we counted the number of thrashes over 30 s. A total of 20-30 worms were prepared per group. The assay was repeated thrice. Two-way ANOVA along with Sidak multiple comparisons test was used to evaluate the *P*-values.

### Avid assay

The age-associated vulva integrity defects assay was performed as described previously [31]. N2 animals were synchronized and raised at 20 °C on NGM plates seeded with OP50 until L4 stage. Approximately 15 animals per plate with a minimum of eight plates per group were transferred to either control or verapamil (100 μM and 400 μM) treatment plates containing 50 μg/mL 5-Fluorodeoxyuridine (FudR). Animals were periodically transferred to avoid contamination and to prevent starvation. The number of Avid in each plate was recorded once daily throughout the whole life of animals. Experiments were conducted thrice. Unpaired t-test was used to assess the *P-*values.

### Osmotic stress resistance assay

The osmotic stress resistance assay was performed as described previously [56]. Approximately 60 animals administrated with either control or verapamil (100 μM and 400 μM) for 6 days were placed on 500 mM NaCl containing NGM plates and their movement (# moving/total) was assessed at 3, 5, 7, 9, 11, and 13 minutes. Experiments were repeated at least thrice. The log-rank (Mantel-Cox) test was used to evaluate the curve significance.

### Cell culture

MRC-5 cells were cultured in MEM (Gibco) supplemented with 1% nonessential amino acid solution (BI), 10% fetal bovine serum (Gibco), 1% penicillin/streptomycin solution (Yeasen), and 1% sodium pyruvate solution (BI). Cells were maintained in an incubator at 37 °C under 5% CO_2_.

### Cell viability assay

A cell counting kit 8 (CCK8) assay was used to assess the MRC-5 cell viability. Cells were seeded into a 96-well plate at 5 × 10^4^ cells per well, and treated with varying concentrations of verapamil (0.39 μM, 0.78 μM, 1.56 μM, 3.12 μM, 6.25 μM, 12.50 μM, 25.00 μM) for 48 h or 72 h. Then, CCK8 solution was added to the wells of the plate, which was then incubated at 37 °C for 2 h. Then, the absorbance of each well was recorded at 450 nm using a Microplate Reader (Bio-Tek Instruments, Synergy, H1). Cell viability (%) was evaluated according to the following equation: (average absorbance of the treatment group/average absorbance of the control group) × 100%. The assay was performed thrice. Unpaired t-test was used to evaluate *P-*values.

### SA-β-Gal staining assay

SA-β-Gal staining assay was conducted using a Senescence β-Galactosidase Staining Kit (Beyotime), according to the manufacturer’s instructions. Cells were treated with verapamil (3 μM,) for 3 days, while metformin used a positive control. Then, cells were washed using PBS and fixed in 4% formaldehyde and 0.2% glutaraldehyde for 15 min at room temperature. Then, the fixed cells were stained using fresh staining solution and incubated at 37 °C overnight to assess SA-β-Gal activity. Before imaging under Nikon Eclipse Ts2R inverted microscope at 100× magnification, the cells were stained with DAPI (Sigma). SA-β-Gal-positive cells were quantified. Unpaired t-test was used to evaluate the *P-*values.

### Pharyngeal pumping assay

Wild-type N2 *C. elegans* were cultured on NGM according to the lifespan assay protocol. For control and verapamil (100 μM and 400 μM) treatment group, 20-30 worms were prepared for the pharyngeal pumping assay. On days 3, 6, 9, 12, and 15, the pharyngeal pumping rate was evaluated by quantifying the contractions of the pharynx over a period of 1 min. The assay was performed thrice. Two-way ANOVA along with Sidak multiple comparisons test was used to evaluate *P*-values.

### Dietary restriction

From day 0 of the lifespan assay, dietary restriction was carried out through bacterial dilution as already described [57]. An overnight culture of *E. coli* OP50 was centrifuged at 3,000 rpm for 30 min. Bacterial cells were diluted in S buffer to concentrations of 1 × 10^9^ CFU/mL (for DR) and 1 × 10^10^ CFU/mL (for AL). Then, bacterial cells were seeded on NGM plates treated with control or verapamil (400 μM) for the lifespan assay, while carbenicillin (50 mg/mL) was contained in all NGM plates.

### Calcineurin activity assay

Worms were grown after a lifespan assay, and a sufficient number of worms were prepared. On day 6, total protein of control and verapamil (100 μM) treated group was extracted from the worms using RIPA buffer supplemented with phosphatase and protease inhibitors and quantified using a BCA Protein Quantification Kit (Yeasen). Then, calcineurin activity was measured using a calcineurin ELISA Kit, according to the manufacturer’s instructions. An unpaired t-test was used to assess *P*-values.

### RNAi experiment

A bacterial feeding RNAi experiment was carried out as described previously [58]. *E. coli* strain HT115 was used for this assay. The clone used was *tax-6* (C02F4.2). L4440 was used as the vector. Worms fed with bacteria expressing L4440 or engineered to produce a *tax-6* RNAi effect were cultured until the F4 stage, and one subset of the worms was confirmed to exhibit decreased expression of the *tax-6* gene via qPCR. Then, the other subset of worms was synchronized for a lifespan assay on control and verapamil (100 μM) treated NGM plates seeded with bacteria either expressing L4440 or engineered to produce a *tax-6* RNAi effect.

### Western blot analysis

MRC-5 cells were either or not treated with either 3 μM or 15 μM verapamil for three days. The protein sample from each group was analyzed using 12% tris-glycine SDS PAGE, and transferred to PVDF membrane. The membrane was blocked using 5% nonfat dry milk for 1 h at room temperature, followed by overnight incubation at 4 °C with antibodies against target proteins: LC3B (ab192890, 1/2000 dilution, from Abcam) and β-actin (sc-47778, 1/1000 dilution, from Santa Cruz). Next, the membrane was incubated with species-specific HRP-conjugated secondary antibody, followed by chemiluminescence substrate. The membrane was then subjected to imaging techniques. ImageJ 1.50 software was used to quantify the band intensity.

### Analysis of autophagic events using an LGG-1 reporter strain

The degree of *C. elegans* autophagy was monitored using a GFP::LGG-1 translational reporter as described previously [59]. At L3 stage, the GFP-positive puncta in the seam cells of the worms was quantified using a Leica confocal microscope (Leica TCR 6500) at 630× magnification. Synchronized eggs were moved to control and verapamil (100 μM) treatment plates seeded with OP50 cells, bacteria expressing L4440, or bacteria engineered to produce *tax-6* RNAi effects. Then, GFP::LGG-1-positive puncta were counted at the L3 stage. At least 3-10 seam cells from each worm were examined under at least two independent trials and the results were averaged. The average value was used to calculate the mean population of GFP::LGG-1-containing puncta per seam cell. An unpaired t-test was used to assess the *P*-values.

### Quantitative real-time PCR

Synchronized worms were grown on NGM plates seeded with OP50. At L4 stage, worms were transferred to NGM plates either or not containing verapamil (100 μM) and raised for 6 days. Then, their RNA was extracted. For *tax-6* RNAi experiment, synchronized worms were grown on NGM plates seeded with *tax-6* RNAi bacteria or L4440 to F4. RNA Extraction Kit (Omega) was used to extract the RNA, from the adults after 3 days, which was then reverse transcribed using cDNA Synthesis Kit (Yeasen). An RT-qPCR SYBR Green Kit (Yeasen) was used to perform qPCR.

## Supplementary Material

Supplementary Materials and Methods

Supplementary Figures

Supplementary Tables
