# Supplementary material for: Verapamil extends lifespan in Caenorhabditis elegans by inhibiting calcineurin activity and promoting autophagy
Source: Aging (Albany NY). 2020 Mar 24;12(6):5300–17. doi: 10.18632/aging.102951 (PMC7138547; doi:10.18632/aging.102951)
Supplement: Supplementary Materials and Methods [file aging-12-102951-s002..pdf]

SUPPLEMENTARY MATERIALS AND METHODS

Heat stress resistance assay

The heat stress resistance assay was performed as described previously [1]. Approximately 150 animals administrated with control or verapamil (100 μM and 400 μM) for 6 days were incubated at 35 °C for 24 hours. The number of surviving animals was counted every 3 hours after 8 hours. Experiments were repeated thrice. The log-rank (Mantel-Cox) test was used to assess curve significance.

D. melanogaster lifespan assay

D. melanogaster lifespan assays were performed as discribed [2], and the strain W^{1118} was used. Synchronized flies were collected under mild CO_2 anaesthesia and distinguished female or male. Then males were transferred to diets of control and verapamil (50 μM)-treated groups. The number of flies was counted every other day. Flies were transfered to fresh diets every other day. GraphPad Prism 6 was used to construct the survival curve and the log-rank (Mantel-Cox) test was used to assess curve significance.

CRISPR/Cas9 technology for generation of *hlh-30* mutation

Two sgRNAs targeting the coding sequence of *hlh-30* were designed by the Guide Design Tool (http://crispr.mit.edu). Synthesized sgRNA fragments were inserted into the pDD162 vector (Addgene #47549). The resultant Cas9-sgRNA plasmids (50 ng/μL) were co-injected along with selection markers pCFJ90 *Pmyo-2*::mCherry (1 ng/μL) and pRF4 *rol-6* (su1006) (50 ng/μL) into N2 cells. The F1 progeny were examined by PCR and sequencing for indel mutations. The *hlh-30(hq293)* worms harbor one-nucleotide deletion within the exon, which leads to premature stop codons within the coding sequence of the BHLH domain in all the transcripts of *hlh-30*. sgRNAs for hlh-30 mutation: TGTTCAGGTCGTCTCAAGTT; TCAA TGTCGATCGAACTCGT; Sequence variation of hlh-30; >WT: agcagtatgataaaaatgaccatgtgccttgaaaattgataca ataagtgttatatcgaacgaaggaacgaaacaaaaaaaaccggtttctcatca gatcctcctcctactttccgtcgattttgcgccaaaaattgtctctctaatttctcaa gttatatgccccaaaatgttcagGTCGTCTCAAGTTCGGCGC CGACGAGTTCGATCGACATTGAGAAGATGATT GGCGCCGTGTCGAACGGCGGTGGGAATAGTGG CGGTGATAATGATCCGGAGGACTATTACCGCG ACCGCAGGAAGAAGGACATTCACAATATGAgtga gttttcgaggctttcaaattttttaaaatgaattttcgattcatttttttcagTTGA ACGCCGACGAAGATATA...

>hq293: agcagtatgataaaaatgaccatgtgccttgaaaattgataca ataagtgttatatcgaacgaaggaacgaaacaaaaaaaaccggtttctcatca gatcctcctcctactttccgtcgattttgcgccaaaaattgtctctctaatttctcaa gttatatgccccaaaatgttcagGTCGTCTCAAGTTCGGCGC CGAC-AGTTCGATCGACATTGAGAAGATGATT GGCGCCGTGTCGAACGGCGGTGGGAATAGTGG CGGTGATAATGATCCGGAGGACTATTACCGCG ACCGCAGGAAGAAGGACATTCACAATATGAgtga gttttcgaggctttcaaattttttaaaatgaattttcgattcatttttttcagTTGA ACGCCGACGAAGATATA...-: a frameshift mutation that leads to multiple early stop codons.

DAF-16::GFP translocation assay

DAF-16::GFP translocation into nuclei was visualized using Nikon Eclipse Ts2R inverted microscope at 100× magnification. DAF-16 localization was classified into cytosolic, intermediate, and nuclear localization [3]. The number of animals with each level of translocation was counted after administration of control or verapamil (100 μM) for 6 days. Experiments were conducted twice.

HLH-30::GFP translocation assay

HLH-30::GFP localization into nuclei was visualized using Nikon Eclipse Ts2R inverted microscope at 100× magnification. Over 30 animals per group were imaged after 6 days of control and verapamil (100 μM) treatment. Experiments were repeated thrice. An unpaired t-test was used to calculate the *P*-values.

REFERENCES

1. Chen D, Thomas EL, Kapahi P. HIF-1 modulates dietary restriction-mediated lifespan extension via IRE-1 in Caenorhabditis elegans. PLoS Genet. 2009; 5:e1000486. https://doi.org/10.1371/journal.pgen.1000486 PMID:19461873
2. Slack C, Foley A, Partridge L. Activation of AMPK by the putative dietary restriction mimetic metformin is insufficient to extend lifespan in Drosophila. PLoS One. 2012; 7:e47699. https://doi.org/10.1371/journal.pone.0047699 PMID:23077661
3. Schafer JC, Winkelbauer ME, Williams CL, Haycraft CJ, Desmond RA, Yoder BK. IFTA-2 is a conserved cilia protein involved in pathways regulating longevity and dauer formation in Caenorhabditis elegans. J Cell Sci. 2006; 119:4088–100. https://doi.org/10.1242/jcs.03187 PMID:16968739
