# Supplementary material for: Verapamil extends lifespan in Caenorhabditis elegans by inhibiting calcineurin activity and promoting autophagy
Source: Aging (Albany NY). 2020 Mar 24;12(6):5300–17. doi: 10.18632/aging.102951 (PMC7138547; doi:10.18632/aging.102951)
Supplement: Supplementary Figures [file aging-12-102951-s003..pdf]

SUPPLEMENTARY FIGURES

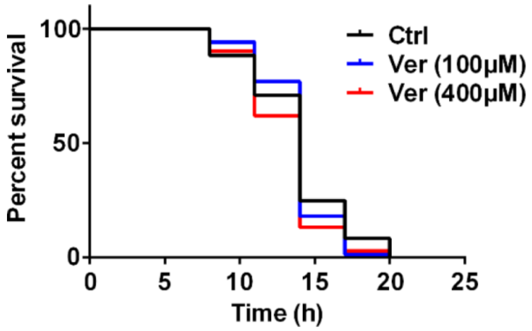

Supplementary Figure 1. Verapamil does not increase heat stress tolerance in *C. elegans.* Verapamil (100 µM, 400 µM) did not improve heat stress tolerance in *C. elegans*. The log-rank (Mantel-Cox) test was used to calculate the *P*-values.

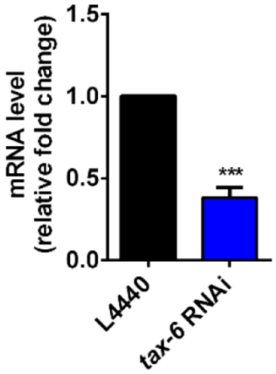

Supplementary Figure 2. *tax-6* gene mRNA level after *tax-6* RNAi treatment. The mRNA level of *tax-6* decreased after RNAi treatment ($^{***}P < 0.001$). An unpaired t-test was used to evaluate the *P*-values and error bars represent SEM.

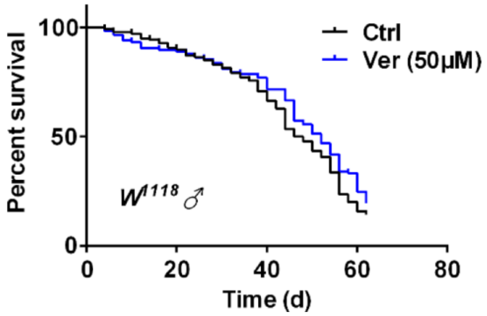

Supplementary Figure 3. Verapamil extends the lifespan of male *D. melanogaster*. Verapamil (50 µM) extends the lifespan of male *D. melanogaster*, but not significantly, possibly because the dose of verapamil used was too high for male *D. melanogaster* that could have led to some toxicity. The log-rank (Mantel-Cox) test was used to calculate the *P*-values.

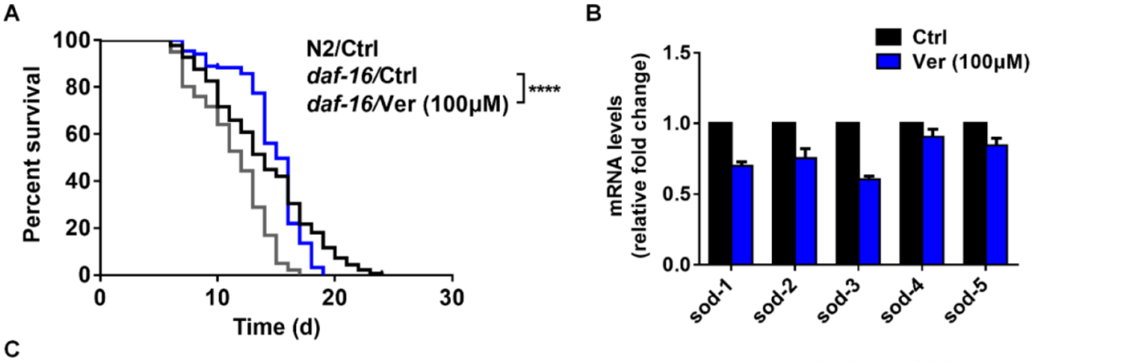

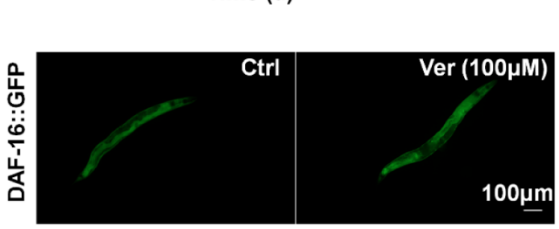

	DAF-16::GFP worms (%)	
	Ctrl	Ver (100 μM)
Cytoplasmic	22 (79)	31 (100)
Intermediate	6 (21)	0 (0)
Nuclear	0 (0)	0 (0)
Total	28 (100)	31 (100)

Supplementary Figure 4. Verapamil-mediated lifespan extension is DAF-16-independent. (**A**) Verapamil (100 μM) extended *daf-16* mutant lifespan (*****P* < 0.0001). The log-rank (Mantel-Cox) test was used to calculate *P*-values. (**B**) Verapamil (100 μM) did not increase the expression of *sod* family. Multiple t-tests were used to assess the *P*-values and error bars represent SEM. (**C**) Verapamil (100 μM) treatment did not lead to nuclear translocation of DAF-16::GFP.

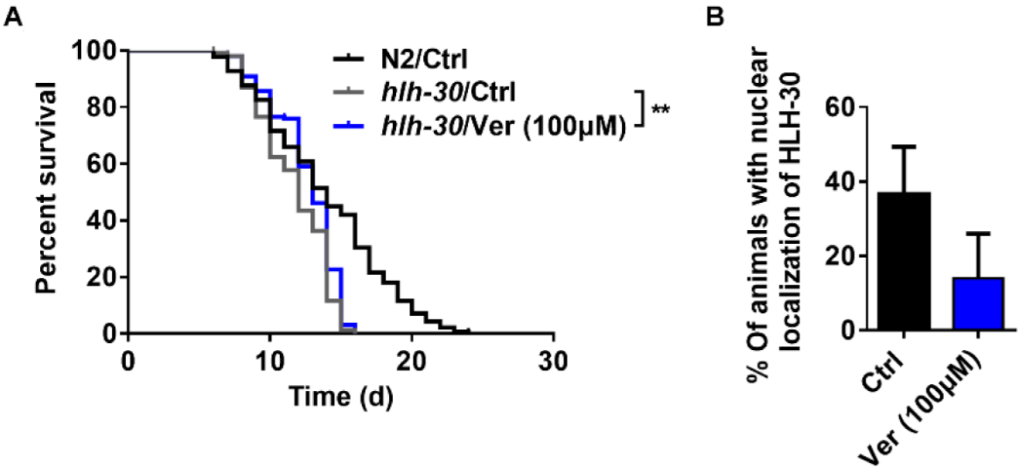

Supplementary Figure 5. Verapamil-mediated lifespan extension is HLH-30-independent. (**A**) Verapamil (100 μM) still extends the lifespan of *hlh-30* mutant (***P* < 0.01). The log-rank (Mantel-Cox) test was used to calculate *P*-values. (**B**) Verapamil (100 μM) treatment did not lead to nuclear translocation of HLH-30::GFP. An unpaired t-test was used to calculate the *P*-values and error bars represent SEM.
