# Supplementary material for: Verapamil extends lifespan in Caenorhabditis elegans by inhibiting calcineurin activity and promoting autophagy
Source: Aging (Albany NY). 2020 Mar 24;12(6):5300–17. doi: 10.18632/aging.102951 (PMC7138547; doi:10.18632/aging.102951)
Supplement: Supplementary Tables [file aging-12-102951-s001..pdf]

SUPPLEMENTARY TABLES

Supplementary Table 1. Lifespan data of male *D. melanogaster.*

Strain	Median lifespan (days)	Mean lifespan (days)	Number of flies	*P*-Value
W^{1118}/Ctrl	47	41.90	120	—
W^{1118}/Ver (50μM)	52	42.72	94	0.0984

Supplementary Table 2. Lifespan data of *C. elegans*.

Strain	Drug treatment	Mean lifespan (days)	Maximum lifespan (days)	Number of worms	*P*-Values
N2	—	13.94	24	138	—
daf-16 (mu86)	—	11.37	17	142	—
daf-16 (mu86)	Ver (100μM)	14.63	19	155	<0.0001
hlh-30 (hq293)	—	11.74	16	154	—
hlh-30 (hq293)	Ver (100μM)	12.58	16	154	<0.01
